# Observations upon Otorrhea as a Sequela to Scarlet Fever

**Published:** 1858-06

**Authors:** Laurence Turnbull

**Affiliations:** Physician of the Eye and Ear Department of the Western Clinical Infirmary of Philadelphia


### SELECTIONS.

*Observations upon Otorrhœa as a Sequela to Scarlet Fever.* By Laurence Turnbull, M. D., Physician of the Eye and Ear Department of the Western Clinical Infirmary of Philadelphia.

Otorrhœa, or a chronic discharge from the ear, is one of the most frequent and tedious affections which the physician has to treat. It may properly be divided into two great classes, depending on the nature of the discharge, viz: mucous and purulent. The mucous form arises as a termination of catarrhal otitis, from measles, or whooping-cough. Its chief characteristic is the absence of true pus from the discharge, and its being found much more amenable to treatment than the purulent variety, which we design to treat in this paper. Occasionally we find this second form of discharge difficult to manage in scrofulous patients, when neglected; but if proper counter-irritation be made behind the ear, and great cleanliness of the ear and skin be enforced, with tonics, astringents, and slightly drastic purgatives, it will be found more amenable to treatment than the form depending on scarlet fever. The great importance of attention to this second form of otorrhœa (chiefly the result of scarlet fever,) will be better understood, when I state that it is the chief cause of non-congenital deafness. This is proven by the records of the deaf and dumb institutions, both of this country and of Europe.* An examination of the reports of the cases admitted into the Pennsylvania Institution for the Deaf and Dumb, in the City of Philadelphia, during the last six years, also proves the same fact. In 1852, of the thirty-three pupils admitted, seventeen were born deaf, three lost their hearing by scarlet fever, the remainder from five different causes, and four from causes unknown.

For the year 1853, of the twenty-six pupils admitted, twelve were born deaf, six lost their hearing by scarlet fever.

In 1854, of the forty-seven pupils admitted, thirty-two were born deaf, three lost their hearing by scarlet fever.

In 1855, of the twenty-five pupils admitted, thirteen were born deaf, five lost their hearing by scarlet fever.

* See Wilde on Diseases of the Ear.

In 1856, "of the sixty-three pupils admitted, 27 were born deaf, 12 lost their hearing by scarlet fever. The large number for 1856 may be accounted for, by the fact that in that year the mortality from scarlet fever in Philadelphia was nine hundred and seventy-two in a population of 500,000, while in 1855, the deaths from scarlet fever were only 163. This fever also prevailed throughout the State as an epidemic, during the latter part of the year 1855 and 56.

Otorrhœa, resulting from scarlet fever, is a disease which, if it becomes purulent and chronic, is very difficult to cure. This, I find to be the opinion of almost every one who has devoted much attention to the subject. Itard* says: "It is of interminable duration, and is one of the gravest diseases of the organs of hearing." Mr. Wilde† remarks: "The most unmanageable cases of otorrhœa which I have met with in practice, in which the most destruction has taken place, and where the ossicula have been most frequently lost, have been the result of scarlatina." In my own practice, one case had existed for thirty-five years, in another twelve years, and a third case, which came under my care, had been more or less under medical and surgical treatment, for seven years. In several other cases, the duration was respectively, one, two, and three years. The most recent cases have been the result of the late epidemic in our city. The profession has not, at any time, been sufficiently alive to the great importance of the treatment of acute inflammation of the tympanum, so as to prevent, if possible, the destruction of the internal portion of the ear, and the subsequent otorrhœa.

If the same amount of care and trouble were exercised by physicians in the treatment of the *ear* as of the throat, a much smaller number of children would be permanently deaf, and a much smaller number would suffer from destruction of the tympanum, or with chronic otorrhœa for months or years after. Having been successful in all my cases, during the late epidemic, in preventing deafness, I will give an outline of my treatment, hoping it may save a few children that important organ, the ear, so adapted to increase knowledge, and delight mankind.

*Treatment.*—In the early stage, when the scarlet fever is at its height, we must endeavor to arrest the acute inflammation of the ear, by depletion, but care must be exercised, as this exanthem will frequently assume a low type, which was the case during the recent epidemic. In such cases, local depletion (by leeches or small cups, to the mastoid process and antitragus) should be employed as soon as acute pain is complained

* Traite des Maladies de l'Oreille et de l'Audition. Par J. M. G. Itard, p. 203.

† Wilde, p. 323.

of, and sometimes it will be found necessary to make pressure, as the child may be too young to indicate the point of pain, except by sudden screaming and crying; but pressure at the lower portion of the ear will reveal the cause instantly.

Local depletion should be repeated at intervals, and in such quantities as the strength of the child will permit, assisted by active purgation by a drastic agent, as jalap, scammony, or senna, in infusion, while, at the same time, we support the child's strength with nourishing diet, &c.

If the case will not bear depletion, or we are called too late, then we must still apply counter-irritation, and purge the patient, but should suppuration have commenced, indicated by a chill, with increased pain, of a darting and throbbing nature, with a sense of bursting in the ear, the meatus, on examination, being of a livid red color, with the membrane of the tympanum red and swollen, our proper plan is to introduce a delicate cataract needle and puncture the membrane. This will liberate the fluid; the purulent matter being pent up in the tympanum, from which it cannot escape through the Eustachian tube, it may ulcerate its passage externally, or may, by its contact, cause destruction of the internal ear, with destruction of the tympanum by rupture, or even extend towards the meninges of the brain, being not only fatal to the organs of hearing, but even to life itself. Cases are on record, in which this ulcerative process has extended, so as to open the carotid artery into the Eustachian tube, causing death from hemorrhage from the ear. Or the extension to the brain may be in the form of effusion or disease of the periosteum and death from convulsions. But instead of this extension your remedies may have prevented death, and the disease may now take on the subacute form attended with a discharge of a muco-purulent or sero-purulent matter The treatment in such cases must be both general and local. The general treatment, which is of the utmost importance, is to improve the blood by tonics of iron, quinine, and cod-liver oil, with the frequent use of the bath, or wet towel, with frictions, and out-door exercise in clear weather. The local treatment should be directed to the throat, by the application of solid nitrate of silver to the region of the Eustachian tube, every third day, with stimulating gargles, and the internal use of weak astringent washes to the ear, while most active counter-irritation should be kept up by blisters, setons, or croton oil, applied over the mastoid region and tonsils. In some of the most unpromising cases the otorrhœa will gradually cease, and the disease may thus be cured; and if the case becomes chronic, then the treatment must be continued for months, and even years, as may be seen by the record of a few cases.

CASE I. *Scarlatina, Loss of Membrana Tympani, Deafness, Otorrhœa.—July* 11, 1856. John B., æt. 10, had scarlet fever at the age of three; deafness has ever since prevailed, with otorrhœa on both sides; the external ear in normal condition; meatus, good size; membrana tympani quite gone on right side, while on the left it is thickened with a small opening in its centre.

He is pale, of scrofulous diathesis, with several enlarged cervical glands; nose and throat in an irritable condition. Able to pass air through both Eustachian tubes.

*Treatment.*—After washing and drying out the meatus, and opening in left membrana tympani, it was brushed over with a solution of nitrate of silver every third day, while internally was administered cod-liver oil and ½ grain of sulphate of quinia in solution three times a day; counter-irritation behind the ear by tincture of iodine.

*January*, 1857. After six months' treatment, the otorrhœa on left side is quite gone, and the opening filled up, while the discharge from the right is very slight, unless on exposure to cold air, which is much obviated by the introduction of a pellet of cotton, and avoidance of cold winds. The hearing was so much improved that he was able to resume his school duties, requiring no artificial aid.

CASE II.—*July* 22, 1856. David R., æt. five years, had scarlet fever at the age of two years and seven months, membrana tympani in part gone; general health not good, pale, anæmic; cleansing and application of solution of nitrate of silver, with the internal use of sulphate of cinchona, one grain, three times a day; and as a local astringent, gr. v cupri sulphas to ℥j of water; and to moisten the meatus with glycerine, in which was suspended tannic acid.

*Nov.*, 1857. Hearing much improved, and discharge improved. (Case did not return.)

CASE III. *Scarlatina, Loss of Tympani, Deafness, Rheumatism with Polypus.—August* 15, 1856. William J. M., æt. 28 years; otorrhœa from both ears; had scarlet fever at the age of sixteen; could only hear the tick of a watch when applied ever the temporal bone; Eustachian tubes both closed; bent almost double with rheumatism; right ear, membrana tympani entirely gone, and no malleus visible; left ear filled up with a polypus, covered with yellow pus, and flowing out over the edge of the meatus.

*Treatment.*—Two grains of iodide of potassium three times a day, with wash of ten grains zinci sulphas, to be dropped into the left ear. After cleansing with cotton, applied solid nitrate of silver to growths, by means of Wilde's caustic holder. Finding, after several trials during August, not much reduction in size, I twisted off portions, at three different sittings, and, as the hemorrhage was considerable, I applied powdered alum, and again used the nitrate of silver to get rid of what was still at the bottom, but found it did not entirely destroy the spongy granulations, after eight months' persevering trial.

*Feb'y*, 1857. Applied a saturated solution of zinci chlorid., on a piece of cotton, by means of the speculum, for three weeks, with the entire contraction of the granulations, so that now, I can see the membrana tympani, and an orifice from which the whole mass of granulations seems to have sprouted, and as I touch the surface, he feels a disposition in his throat to cough. His general health is much improved, being able to straighten himself, and rheumatism gone.

*March*, 1857. Dilated the Eustachian tube by means of Wild's catheter and warm air twice, at two weeks' interval. His hearing is so much improved that he can hear me speak to him at a distanee of six or seven feet. Gradually the membrana tympani closed over, and he was discharged, cured.

This case was one of great interest, and shows the good results to be obtained by persevering efforts, assisted as it must be, by the willing help of the patient, and the use of constitutional remedies.

CASE IV. *Otorrhœa, of twelve years' duration, of both Ears, with Perforation of Membrana Tympani on one side: Improved.—April* 15, 1857. Benj. B. F., æt. 24; a healthy looking young man, of fair complexion and blue eyes. When he speaks, it sounds as if there was some obstruction in the throat or nose. (Blacksmith.) He stated that he had scarlet fever at the age of about twelve, but of a mild character. On getting well, he had more or less discharge from both ears, and was deaf, and although he has been under the care of several physicians, he has become worse, so that he is unable to attend properly to his duties in his father's shop. On examination with Wild's speculum, found the right membrana tympani with a small opening in it, but he was not able to pass air through it, from obstruction of the Eustachian tube; there was also redness of the external meatus; hearing distance one inch; left ear, discharge, with redness, and in places, whiteness of the membrane, but no perforation; able to inflate the membrane on this side; hearing distance three inches. Throat—both tonsils very much enlarged, but not very hard; relaxation and elongation of uvula, with engorgement. Removed uvula, made application of zinci chlorid., in solution, to tonsils, and directed cleansing of the ear by a syringe, and application of solution of zinci sulphas, with vin. opii gr. x to three ounces of distilled water, three times a day. For internal use, syr. sarsap. comp., with gr. j bichl. hydrarg., a wineglassful three times a day, with nourishing diet.

22*d*. Throat of a much better color, and speaks plainer; not much improved in discharge from ear, which annoys him, having to wipe it very frequently through the day, as I directed him to keep no cotton in the ear, but to wash it frequently each day with the wash diluted.

Treatment of throat continued; applied cantharidal collodion below and in front of the ear, with application of x gr. solution of argenti nitratis to ear, with insufflation of powdered alum to throat and nose.

25*th*. Very much improved. Ordered him solution of iodine comp., ten drops three times a day, in infusion of hops.

*May* 16. Finding the Eustachian tube continued obstructed, introduced Eustachian catheter, and gently dilated it with warm air, which produced a feeling of faintness, but which soon passed off. Same treatment continued.

23*d*. Catheter again introduced, no feelings of faintness being produced. Same treatment.

27*th*. Both ears discharge less. Eustachian tube open, the air passing through it into the middle ear, and being discharged from thence outwards, by the perforated membrana tympani. Hearing improved.

*June* 3. Had a slight relapse from catarrh; throat more congested; discharge from ear increased; applied solution to the throat, also mild solution to the ear; directed demulcents to be used.

20*th*. Again improving, but is feeble. Ordered tonics, sulphate of quinine, and cod-liver oil.

*July* 11. Discharge ceased; complains of pain in his head. Ordered blisters behind ears, and an infusion of senna, to keep up action on the bowels, so as to prevent a return of the pain.

*August*. Called again at the infirmary, and informed me he was so much improved as to be able to take his place as superintendent of the shop, and when on a recent visit to the river, he could hear the noise of the steamboats, which he had not been able to do for many years.

Case V. *Otorrhœa of both Ears of five years' duration, with loss of both Membrana Tympani.*—The result of scarlet fever and measles. Mary T., æt. 8 years, a delicate dark-eyed girl, is deaf in both ears, with great irritation from profuse purulent discharge, the odor from which is very offensive. Has tried numerous physicians without success. After the acute attack, the father was directed by his physician to syringe the ears, every few days, with tepid water, which he continued, until one day the discharge came through the nose, and the child cried out that something had burst, since which time numerous applications have been tried, but with no permanent benefit.

On a careful examination and removal of the profuse secretion by pellets of cotton, I found both membrana tympani entirely gone, the discharge proceeding from the middle ear. The tonsils were enlarged, the throat red and congested, with several enlarged cervical glands.

I directed simple cleansing of the ear with an infusion of chamomile flowers, several times a day, with a weak lotion of subacetate of lead, to reduce the irritation of the auricle, to be applied with a pledget of lint. Also, a solution of zinci sulphas gr. j to the ℥j of water, applied by means of a small syringe, and then to allow it to flow out.

Directed, also, that her diet should be nourishing, with the use of cod-liver oil, a teaspoonful three times a day, with a salt bath once a week.

*March* 24. To-day, offensive discharge much less, right ear much improved in appearance. Directed ½ gr. of sulphate of quinia three times a day, with the cod-liver oil to be continued.

*April* 8. Much improved in all respects; discharge still continues; was able to resume school duties.

Case VI. *Cephalic Otorrhœa the result of Scarlet Fever of three years' duration; Post-mortem.*—Mary H., æt. six years; had scarlet fever severely when three years of age. Was a long time feeble, but her ear was not treated, except to wash and keep it clean.

Towards May, 1857, the child began to complain very much, and came to my office. Ordered a small blister, with a solution of one gr. sulphate of zinc to one ounce of water. As an internal tonic ½ gr. sulphate of quinia three times a day. On examination, the right membrana tympani was gone, and there was considerable discharge of unhealthy pus from the ear, showing disease of the bones. Having been benefited by the treatment, she did not return to the office until June 6th, when she was suffering intense pain from catarrh in her head, the discharge being much increased from exposure; being a very wilful child, the parent had but little control over her. I directed counter-irritation to be renewed, with the internal use of an opiate, to relieve pain; did not leech her on account of her feeble state.

Visited her on the 8th. Pain still very persistent, and head bent to the side of the affected ear. Fearing convulsions, ordered four leeches to the back of each ear, with sedatives internally, opiate fomentations to the ear, and warmth to the feet, baths, &c. In spite of treatment she continued to grow worse, and had a severe attack of convulsions on June

26th, which yielded to leeching and cold applications, but was soon followed by a state of coma.

She died on the 18th of June, but previously had considerable discharge of pus from her nose, and was unable to swallow for a day or two before convulsions set in.

The family being very unwilling to allow an examination, I was only permitted to remove the temporal bone and ear, which I did by sawing a V shaped piece, and removing the ear entire. The coverings of the brain, in spite of her anæmic condition and her inability to take nourishment, were much congested and thickened, with effusion of fluid in the ventricles, with considerable softening of the substance of the brain.

*Ear.*—The membrana tympani was almost gone, but, strange to state, in the middle ear, although filled up with green pus, and the membrane soft and detached, I found the malleus and incus, which I have in my collection, but on cleansing the two bones they were found ulcerated. The semicircular canals and cochlea were almost free from disease, which had passed from the middle ear to the membranes and brain, causing a low form of inflammation, with softening and effusion, ending in convulsions and death. What seemed very remarkable in this case, was the long period which the small bones remained in the ear in spite of the discharge, showing how slowly the ligaments which attach the bones ulcerate; thus accounting for the power of hearing in many persons suffering from otorrhœa; the chain of bones, although ulcerated, yet retaining their place, and communicating the sound to the nervous expansion of the auditory nerve. She had no symptoms during life, of facial paralysis; the bending of the head to one side seeming to indicate some disease of the top of the vertebræ, but no examination of that part was permitted by the family.

This imperfect paper on otorrhœa, as a result of exanthematous inflammation of the mucous lining of the tympanal cavity, or extension of the same affection from the throat, upwards through the Eustachian tube, I am well aware is not arranged according to the anatomical classification of Mr. Toynbee, the most distinguished authority on this subject, for although I consider his a good one, yet it has its defects in its extreme subdivision of tissue affected. It is still doubtful to my mind how he, or any one else, can determine the precise tissue which is affected in chronic discharge from the ear, more especially at a public clinic, for the time afforded for examination of each case is often so short, the history given so imperfect, that it is difficult from the patient's statement to know what was the primary cause of the mischief; for we certainly cannot, with all the light we can throw upon it, state which of the five layers of the membrana tympani is affected, when, perhaps, it has been for years bathed with pus, mucus, or a substance which resembles very much the curdy matter found in scrofulous abscesses.

For those interested, I will here give a synopsis of the class of cases met with in my public clinic at the Western Infirmary.

A discharge from the ear will make one-half of my cases arising from chronic inflammation of the external auditory canal, ulceration of the membrana tympani, or disease of the middle and internal ear. Accumulation of cerumen, causing deafness, or an entire want of it, with inflammation of the glands, occurred in about one-half of all my cases, with or without pain and tinnitus aurium. Then follows, in point of frequency, catarrhal inflammation of the auditory passage or middle ear, with mucous accumulations in the Eustachian tube, forming one-tenth of the whole number.

Polypi and fungoüs excrescences in the auditory passages, or projecting through an ulcerated opening in the perforate membrane. Acute cases of inflammation of the membrana tympani are rare, but chronic cases are numerous, with opacity, thickening, &c., with or without occlusion of the Eustachian tube. This is one of the most difficult diseases to treat, if it has been of long standing.

Eruptive affections of the auricle are numerous, as herpes, eczema, &c.

There are certain diseases of the ear which are very rare, namely, nervous deafness, in which the auditory nerve is alone affected.

I will conclude by the recital of a case of complete perforation of the membrana tympani, in which there being no hope of restoring the natural membrane, an artificial one was substituted with benefit to the patient.

Case VII. *Otorrhœa with Perforation of the Membrana Tympani, with the Application of an Artificial Membrane with Success.*—James Riddle, æt. 24 years, a native of Ireland, by occupation a silk weaver, applied January 21, 1858, at the Infirmary, on account of a troublesome discharge and deafness in his right ear, from scarlet fever, which he had at the age of thirteen. He has become so deaf as not to hear a watch with the right ear, even when placed in immediate contact.

On examination with a good light, the meatus was found filled up with dry mucus, pus, &c., upon removal of which, by careful syringing with warm water, the membrana tympani was found entirely gone, and the lining membrane of the meatus thickened and contracted. Astringent and stimulating injections were now employed to change and alter the secretion, and he was directed five grains of iodide of potassium in an infusion of humuli, three times a day, with counter-irritation behind the ear, by cantharidal collodion, and to cleanse the ear with warm water three times a day.

*January* 23 Parts free from accumulation. After wiping out discharge of pus, &c., a small portion of finely powdered cupri sulphus was blown upon the altered mucous membrane, and a wash of zinci sulphas gr. j to each f℥j aqua, was directed to be employed after washing out the ear.

26*th*. Discharge has moderated, but hearing not improved, not being able to pass air through the Eustachian tube. I therefore carefully introduced the Eustachian catheter at two different sittings, and passed air, after some time, through the discharge, so that I could hear the air bubling by the aid of the otoscope.

29*th*. Same treatment; still improving.

*February* 9. Discharge still less. Tried the moistened cotton, so as to improve the hearing, but it increased the discharge; although removed and cleansed, the itching was so intolerable, that he removed it, being unable to bear it, and again resorted to astringent and stimulating applications to moderate the discharge.

12*th*. Introduced an artificial tympanum of vulcanized India rubber, made by Mr. Kobe, of this city, which gave him no pain, and his hearing was improved.

15*th*. The discharge is again on the increase, and has blackened the silver wire, and caused the India rubber to wrinkle and change its color, so that it was removed, and the ear allowed to rest.

18*th*. Is very comfertable to-day; can hear best in the open air. Introduced the tympanum himself, he finding out the right spot; for if he pushes it too far, it is of no use to him. He has been testing his powers of hearing by a clock.

24*th*. Returned to-day and states that he cannot hear so well; when, upon examination, I found the Eustachian tube blocked up again with mucous accumulations. I again dilated it with a series of injections of warm air; and on filling up the ear with a weak solution of cupri sulphas, by making an effort to swallow, air passed up in bubbles through the liquid. This effort to swallow, with the nose and mouth closed, I told him to make each day when he removed his artificial membrane, so as to keep the tube pervious.

*Conclusion.* —This patient continued to visit the Infirmary twice a week until the 17th of March, when he left for the country, having but slight discharge, just sufficient to moisten the artificial membrane; being able to hear conversation with ease and comfort, and even the ticking of an ordinary clock across a room.—*The Med & Surg Reporter.*

